# Twenty-five years of sentinel laboratory-based surveillance of shigellosis in a high-income country endemic for the disease, Israel, 1998 to 2022

**DOI:** 10.2807/1560-7917.ES.2024.29.31.2400022

**Published:** 2024-08-01

**Authors:** Dani Cohen, Orit Treygerman, Shifra Ken-Dror, Orli Sagi, Merav Strauss, Miriam Parizade, Sophy Goren, Analía V Ezernitchi, Assaf Rokney, Lital Keinan-Boker, Ravit Bassal

**Affiliations:** 1 Department of Epidemiology and Preventive Medicine, School of Public Health, Faculty of Medical & Health Sciences, Tel Aviv University, Tel Aviv, Israel; 2 Central Laboratory, Meuhedet Health Services, Lod, Israel; 3 Clinical Microbiology Laboratory, Regional Laboratory Haifa and Western Galilee, Clalit Health Services, Nesher, Israel; 4 Clinical Microbiology Laboratory, Soroka University Medical Center and the Faculty of Health Sciences, Ben-Gurion University of the Negev, Beer-Sheva, Israel; 5 Microbiology Laboratory, Emek Medical Center, Afula, Israel; 6 Microbiology Mega Lab Rechovot, Maccabi Health Services, Rehovot, Israel; 7 Public Health Laboratories – Jerusalem, Public Health Services, Ministry of Health, Jerusalem, Israel; 8 Israel Center for Disease Control, Ministry of Health, Sheba Medical Center, Ramat-Gan, Israel; 9 School of Public Health, University of Haifa, Haifa, Israel

**Keywords:** Shigella, surveillance, epidemics, person-to-person transmission, antimicrobial resistance, host

## Abstract

**Background:**

*Shigella* is a leading cause of moderate-to-severe diarrhoea worldwide and diarrhoeal deaths in children in low- and-middle-income countries.

**Aim:**

We investigated trends and characteristics of shigellosis and antimicrobial resistance of *Shigella sonnei* in Israel.

**Methods:**

We analysed data generated by the Sentinel Laboratory-Based Surveillance Network for Enteric Pathogens that systematically collects data on detection of *Shigella* at sentinel laboratories, along with the characterisation of the isolates at the *Shigella* National Reference Laboratory. Trends in the shigellosis incidence were assessed using Joinpoint regression and interrupted time-series analyses.

**Results:**

The average incidence of culture-confirmed shigellosis in Israel declined from 114 per 100,000 population (95% confidence interval (CI): 112–115) 1998–2004 to 80 per 100,000 population (95% CI: 79–82) 2005–2011. This rate remained stable 2012–2019, being 18–32 times higher than that reported from the United States or European high-income countries. After decreasing to its lowest values during the COVID-19 pandemic years (19/100,000 in 2020 and 5/100,000 in 2021), the incidence of culture-confirmed shigellosis increased to 39 per 100,000 population in 2022. *Shigella sonnei* is the most common serogroup, responsible for a cyclic occurrence of propagated epidemics, and the proportion of *Shigella flexneri* has decreased. Simultaneous resistance of *S. sonnei* to ceftriaxone, ampicillin and sulphamethoxazole-trimethoprim increased from 8.5% (34/402) in 2020 to 92.0% (801/876) in 2022.

**Conclusions:**

These findings reinforce the need for continuous laboratory-based surveillance and inform the primary and secondary prevention strategies for shigellosis in Israel and other endemic high-income countries or communities.

**Key public health message**

**What did you want to address in this study and why?**

Shigellosis is a diarrhoeal disease caused by *Shigella* bacteria. Even though Israel is a wealthy country, it has a high rate of shigellosis, also known as dysentery. To better understand and tackle this problem, we examined the trends in the disease and its characteristics over the past 25 years using data from a network of laboratories.

**What have we learnt from this study?**

We found that shigellosis is more common in Israel than in the United States and European countries, by a factor of 18–32 times. The main culprit is *Shigella sonnei*, responsible for recurrent outbreaks across the country. Moreover, there has been a worrying rise in *S. sonnei* becoming resistant to certain antimicrobials, increasing from 8.5% in 2020 to 92.0% in 2022.

**What are the implications of your findings for public health?**

Our research highlights the need for more effective prevention of shigellosis in Israel and may offer insights for other regions facing similar challenges. Promoting personal hygiene combined with immunisation with a promising vaccine on the horizon could be a key strategy to break the cycle of shigellosis outbreaks and combat the spread of antibiotic resistance.

## Introduction

*Shigella* is a leading cause of moderate-to-severe diarrhoea worldwide. The greatest burden of shigellosis is in low- and middle-income countries (LMICs) with poor sanitation, resulting in ca 250 million cases and more than 200,000 deaths annually, mostly in children aged < 5 years [1].

Improved sanitation and access to clean water sources may not be sufficient to prevent the transmission of *Shigella* due to its very low infectious dose of only 100–1,000 organisms [2]. This is the reason that around 1.5–2 million cases of shigellosis also occur annually in high-income countries (HICs), mostly among toddlers in crowded communities, travellers, soldiers who serve under field conditions in endemic regions and among men who have sex with men (MSM) [3-7].

Shigellosis is often self-limiting, however, in children aged < 5 years, malnourished individuals, older adults and those with weakened immune systems, antimicrobial treatment is indicated [8,9]. Ciprofloxacin (CIP) is currently recommended as the first line of antimicrobial treatment for severe cases of shigellosis, and ceftriaxone (CTR), together with azithromycin (AZ), are the second line [9]. The sustained increase in multidrug resistance (MDR) of shigellae significantly reduces the role of antimicrobials as essential tools for disease control in the absence of a licensed and effective *Shigella* vaccine [8,10].

Despite being a high-income country [11], Israel has remained highly endemic for shigellosis. Person-to-person and fomite-borne transmission are currently the most common modes of faecal-oral *Shigella* transmission. Food-borne outbreaks may occur while waterborne transmission is rare [4]. Here, we present epidemiologic trends of the disease over the past 25 years, including the recently emerging antimicrobial resistance of *Shigella sonnei*, notably against third-generation cephalosporins, using data generated by an active sentinel laboratory-based surveillance network (SLBSN).

## Methods

### Surveillance

The active SLBSN for bacterial enteric diseases was established in Israel in 1998. The five sentinel community laboratories located across Israel that generated the data for the 25-year surveillance of shigellosis were Clalit Health Maintenance Organisation (HMO) laboratory of HaEmek Medical Center in Afula (A) and Clalit HMO laboratory in Haifa (H), representing the north of the country; Clalit HMO laboratory of Soroka Medical Centre in Beer Sheva (S) serving the population in the south; Meuheded HMO laboratory covering the Jerusalem district (M) and Maccabi HMO laboratory with data from the formerly known Dan district (D), serving the population in the centre of Israel. The data on the isolation of *Shigella* at the sentinel laboratories, along with patient demographic information and isolate characterisation at the Ministry of Health National Shigella Reference Center, were collected by the Israel Center for Disease Control (ICDC). Detection of *Shigella* is always included in the laboratory analysis of stool samples of patients with diarrhoeal diseases. The data collection methods were consistent throughout the entire surveillance period.

The population served by the five sentinel laboratories (surveillance population) and the cases of shigellosis were well defined by age, sex, socioeconomic status (SES) and population group: Jews and others (Jews, non-Arabic Christians and those not affiliated with a religion) and Arabs (Muslims, Arab Christians and Druze). The SES was defined using the socioeconomic residential classification published in 2015 by the Israeli Central Bureau of Statistics (ICBS) and ranged between 1, the lowest, and 10, the highest [12].

According to the data of the ICBS and the HMO to which the laboratories belonged, in 2013, the surveillance population covered 30.8% of the total Israeli population of 8.1 million and closely mirrored the sex and age distribution of the population. There was a slight overrepresentation of the Israeli Arab population group, accounting for 26.2% compared with 19.9% in the general population, as can be seen in Supplementary Table S1. 

Detection of *Shigella*, serogrouping, and antimicrobial susceptibility testing (AST) were performed at the sentinel laboratories using routine microbiological procedures. Confirmation of serogroup and determination of serotypes using monovalent antisera (Shigella Antisera, Denka Seiken, Japan) was performed at the Shigella National Reference Laboratory. In 2020, all five laboratories introduced the direct molecular identification of *Shigella* spp. in stool specimen extracts using detection kits for the ipaH gene (Allplex GI-EB Screening Assay, South Korea) common to all *Shigella* species and to enteroinvasive *Escherichia coli*. Since then, in four of the five sentinel laboratories, all stool samples positive by PCR were cultured to isolate *Shigella* for AST and serogroup and serotype determination.

Until the end of 2019, a case of shigellosis was defined as a person with a positive stool culture for *Shigella* of any defined serogroup. From January 2020 onwards, a case of shigellosis was defined based on PCR positivity. A case was counted only once when *Shigella* of the same serotype was isolated from stool specimens submitted within 1 month after the first isolation. Since 2020, the same exclusion criterion for duplicate results has been employed but based only on PCR positivity.

*Shigella* isolates were tested routinely for antimicrobial susceptibility (AST) at four of the five sentinel laboratories, and here we report the antimicrobial susceptibility (AS) for *S. sonnei* and *S. flexneri* between 2010 and 2022. The Vitek 2 automatic device (bioMérieux, France) was used at three laboratories for AST and the Kirby–Bauer disk diffusion method at one laboratory. The two procedures and the interpretation of results followed the guidelines of the Clinical and Laboratory Standards Institute [13]

### Data analysis

Annual incidence rates and 95% confidence intervals (CI) were calculated by dividing the number of laboratory-confirmed cases of shigellosis during the given year by the size of the catchment population of the sentinel laboratories (total) and of the age groups (< 1, 1–4, 5–14, 15–24, 25–34, 35–44, 45–54, 55–64, > 65 years), sex and population groups. Secular trends in incidence and seasonality were evaluated using Joinpoint regression and interrupted time series analysis employing Autoregressive Integrated Moving Average (ARIMA) models. Proportions, odds ratios (OR) with 95% confidence interval (CI), and univariate and multivariate logistic regression were used to assess the prevalence and correlates of antimicrobial resistance of *Shigella* isolates.

Data analysis was performed using IBM SPSS Statistics for Windows, version 29 (IBM Corp., the United States (US)), Joinpoint Regression Programme Version 4.9.0.0, March 2021 (Statistical Research and Applications Branch, National Cancer Institute, US) and the WINPEPI Computer Programs for Epidemiologists [14].

## Results

### Total and sex and age-specific incidence of shigellosis

**Figure 1:**
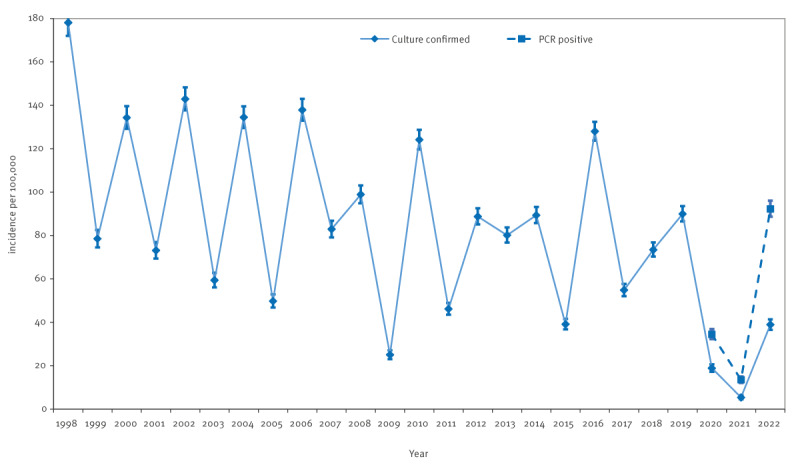
Incidence of shigellosis in the study population, Israel, 1998–2022 (n = 2.5 million)^a^

^a^ Incidence per 100,000 population and 95% confidence intervals, surveillance population covered 30.8% of the total Israeli population of 8.1 million.

The average incidence of culture-confirmed shigellosis in Israel slightly declined from 114 per 100,000 population (95% CI: 112–115) 1998–2004 to 80 per 100,000 population (95% CI: 79–82) 2005–2011 and remained stable 2012–2019 (Figure 1). After decreasing to its lowest values during the COVID-19 pandemic years (19/100,000 in 2020 and 5/100,000 in 2021), the incidence of culture-confirmed shigellosis increased to 39 per 100,000 population in 2022. The corresponding incidence rates of shigellosis, based on PCR-positivity for shigellosis case definition since 2020, were 34, 14 and 92 per 100,000 population in 2020, 2021 and 2022, respectively, 1.8, 2.8 and 2.3 times higher than culture-confirmed shigellosis (Figure 1). Raw data are presented in Supplementary Table S2.

Shigellosis incidence was higher in Jews and others than in Arabs. It decreased in Arabs after 2006 while remaining constant in Jews, as presented in Supplementary Figure S1. The cyclic pattern of disease incidence in the Jewish population closely mirrored the disease pattern in the general population (Figure 1).

The highest incidence peaks occurred in the catchment areas of the M and D sentinel laboratories, as presented in Supplementary Figure S2.

**Figure 2:**
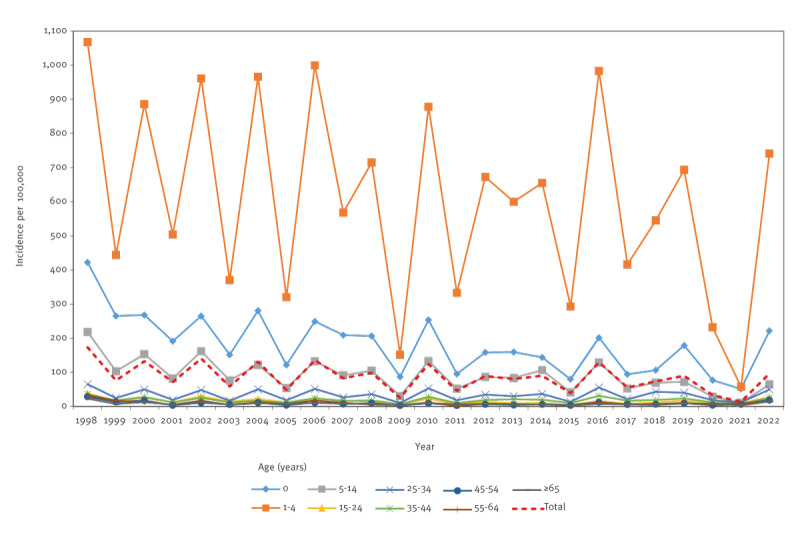
Incidence of shigellosis in the study population, by age, Israel, 1998–2022 (n = 48,887)^a,b^

^a^ Shigellosis cases were defined as culture-confirmed cases 1998–2019 and PCR-positive cases 2020–2022.

^b^ Incidence per 100,000 population, study population covered 30.8% of the total Israeli population of 8.1 million.

In both the total study population and the population group of Jews and others, the incidence rates were highest in the 1–4-year-olds, with 650–1068 cases per 100,000 per year in the peaks of the bi-annual cycles of disease and 300–600 per 100,000 in the years with lower *Shigella* activity with the exception of 2009 and 2020–2021 with the lowest incidence rates, corresponding to years of the swine flu and COVID-19 pandemics, respectively. Children aged < 1 year and the age group of 5–14-year-olds had the next highest incidence rates of shigellosis (Figure 2) and Supplementary Figure S3 for the separate analysis among Jews and others. In the Arab population group, however, between 1998 and 2003, the highest incidence of shigellosis was observed in the first year of life, followed by lower rates in the 1–4-year-olds and lower incidence later in life, as presented in Supplementary Figure S4. Starting in 2004, children aged < 1 year and 1–4 years had similar shigellosis rates.

Shigellosis incidence was significantly higher among males aged < 5 years of both population groups (p < 0.001), except for those aged < 1 year in the population group of Jews and others. Conversely, the female: male risk ratios were similarly > 1 in cases aged > 15 years, as presented in Table S3.

### Secular trends and seasonality of *Shigella* serogroups

**Figure 3:**
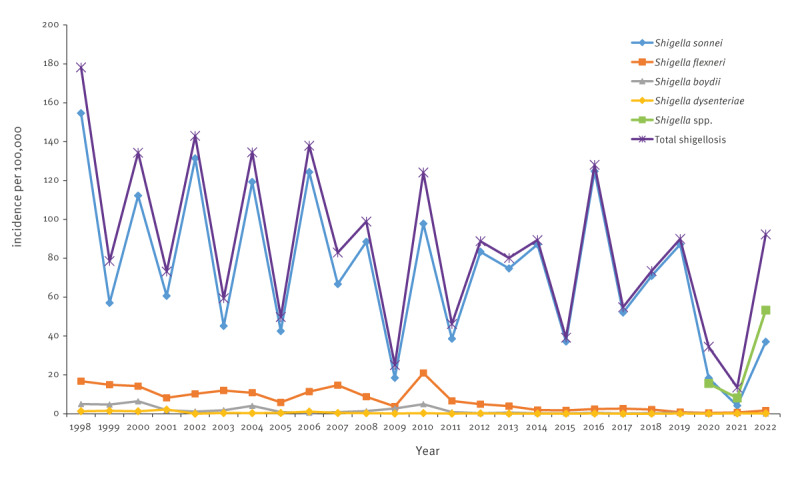
Incidence of shigellosis in the study population, by serogroup, Israel, 1998–2022 (n = 2.5 million)^a^

Figure 3 and Figure S5 illustrate the trend in incidence rates of shigellosis by *Shigella* serogroups. Between 1998 and 2019, i.e. the years with culture-based detection of *Shigella* and before the COVID-19 pandemic years, there was a slight and not statistically significant decrease in total and *S. sonnei* shigellosis modelled incidence rates as demonstrated by the Average Annual Percent Change (AAPC), seen in Figure S5. However, there was a statistically significant decline in the modelled incidence rate of *Shigella flexneri* shigellosis (AAPC = -6.9; 95% CI: -11.0- -2.6; p = 0.004), shown in Figure S5. The incidence of *S. boydii* and *S. dysenteriae* shigellosis was low (Figure 3).

*Shigella* spp.:  positive by PCR but culture-negative; spp.: species; Total shigellosis:  Before 2020, only culture-confirmed cases, 2020–2022, based on ipaH positivity by PCR.

^a^ Incidence per 100,000 population, study population covered 30.8% of the total Israeli population of 8.1 million.

**Figure 4:**
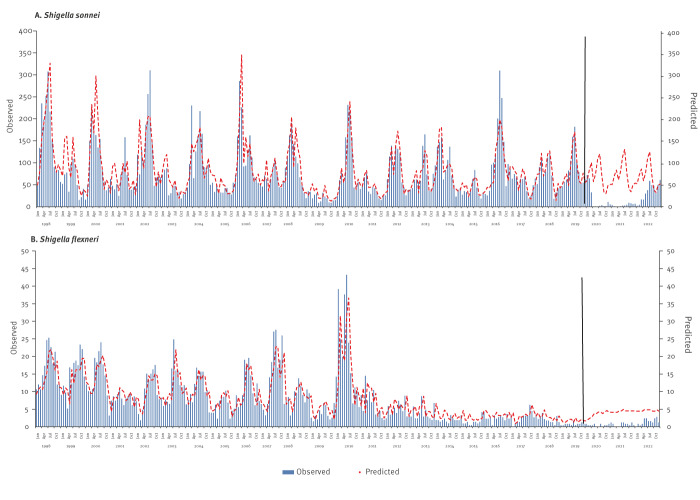
Observed and predicted incidence of infection with *Shigella sonnei* (n = 41,641) and *Shigella flexneri* (n = 3,918), Israel, 1998–2022^a^

^a^ Incidence per 100,000 population, study population covered 30.8% of the total Israeli population of 8.1 million.

The interrupted time series analysis using ARIMA models and monthly incidence data between 1998 and 2019 documented the cyclic and seasonal patterns of *S. sonnei* shigellosis (Figure 4A). This model showed a high stationary R-squared value of 0.880 (p = 0.285) for the Ljung-Box statistic, indicating that the model was correctly fitted with almost complete overlapping of observed and predicted incidences. Moreover, the same model also predicted the counterfactual monthly rates and pattern of *S. sonnei* shigellosis that would have been expected 2020–2022 without the COVID-19 pandemic and related restrictions, including fewer social interactions and improved hygiene measures (e.g. handwashing). The observed monthly rates for these years were substantially lower than the predicted rates (Figure 4A). The ARIMA model based on the 1998–2019 data was also correctly fitted for the *S. flexneri* incidence trend (Figure 4B) (Stationary R-squared value: 0.880; p = 0.285) and could predict the counterfactual monthly rates and pattern of *S. flexneri* shigellosis that would have been expected 2020–2022 without the COVID-19 pandemic and related intervention (Figure 4B).

**Figure 5:**
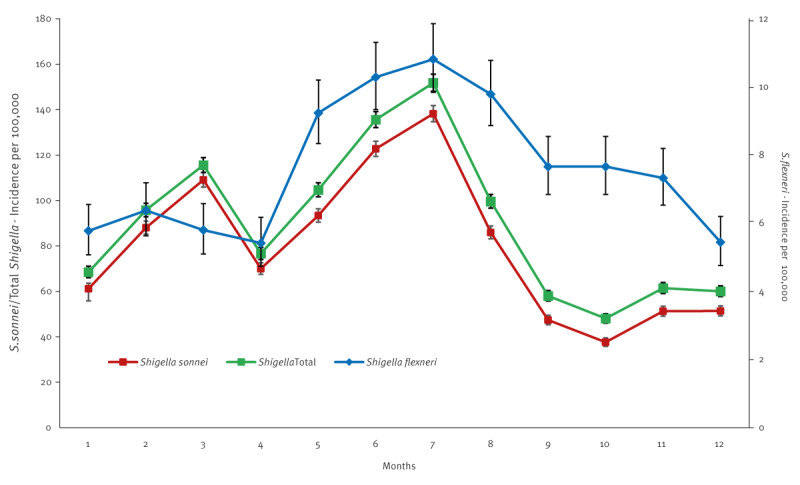
Average monthly incidence of infection with *Shigella sonnei* (n = 40,057) and *Shigella flexneri* (n = 3,837), Israel, 1998–2019^a^

^a^ Incidence per 100,000 population and 95% confidence intervals, study population covered 30.8% of the total Israeli population of 8.1 million.

The ARIMA models also document the bimodal seasonality pattern of *S. sonnei* shigellosis with peaks of disease in March and July and lower rates during the rest of the year and the consistent *S. flexneri* shigellosis highest rates during the summer months, between May and September, declining thereafter. Figure 5 displays the seasonal patterns of *S. sonnei*, *S. flexneri* and total shigellosis as average monthly incidence rates.

The decrease in *S. flexneri* shigellosis was observed in both Jews and others and Arabs, as presented in Supplementary Figures S6 and S7. This led to a significant rise in the relative weight of *S. sonnei* vs *S. flexneri* as the aetiological agent of shigellosis and separately among Jews and others and Arabs, as shown in Supplementary Figure S8. In the study population, 1998–2004 and 2005–2011, 85.0% (n = 13,134) and 84,3% (n = 10,708) of cases of shigellosis, respectively, were caused by *S. sonnei* and 10.8% (n = 1,676) and 12.8% (n = 1,632), respectively, by *S. flexneri*, as presented in Supplementary Figure S8. The corresponding proportions 2012–2019 and 2020–2022 were 95.9% (n = 16,215) and 89.6% (n = 816) for *S. sonnei* and 3.1% (n = 529) and 7.8 (n = 71) for *S. flexneri,* shown in Supplementary Figure S8 (p < 0.001 for the rise in *S. sonnei* and decline in *S. flexneri* isolates for all population groups, between the two periods).

Serotype 6 became the leading *S. flexneri* serotype 1998–2004, 2005–2011 and 2012–2019, accounting for 29.5% (n = 423), 52.8% (n = 848) and 47.5% (n = 172) of the S. *flexneri* serotypes, while *S. flexneri* 2a decreased in its relative weight with 37.6% (n = 539), 21.8% (n = 350) and 17.1% (n = 62) in the same time periods. The small number of *S. flexneri* isolates 2020–2022 limits similar analyses for these years (see Supplementary Figure S9).

### Antimicrobial resistance of *Shigella* isolates

**Figure 6:**
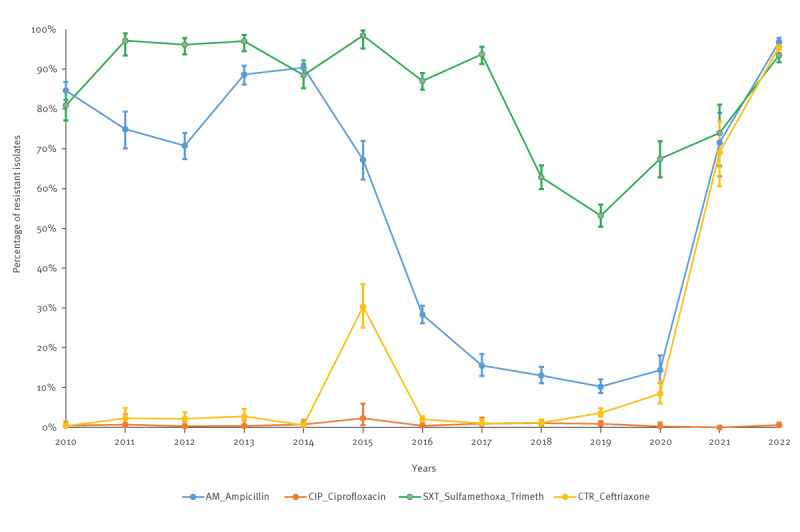
Percentage and 95% confidence intervals of antimicrobial resistance of *Shigella sonnei* isolates, Israel, 2010–2022^a^

^a^ A total of 9,882 isolates were tested for ampicillin, 6,508 for ciprofloxacin, 6,855 for sulfamethoxazole-trimethoprim and 8,469 for ceftriaxone.

Between 2010 and 2022, 9,882, 6,508, 6,855 and 8,469 *S. sonnei* isolates were tested for AMR to ampicillin (AMP), CIP, sulfamethoxazole-trimethoprim (SXT) and CTR, respectively, at the sentinel laboratories. The trends in resistance are depicted in Figure 6 and the raw data are available in Supplementary Table S4.

Between 2010 and 2014, ampicillin resistance varied between 70.8% and 90.4%, declining to 10.2% in 2019, then rising back to 96.8% by 2022. Resistance to SXT was over 90% until 2017, declined to 53.2% in 2019, then increased to 93.5% by 2022. Susceptibility to CTR was high until 2020, with a notable exception in 2015 (30.3% resistance at southern Israel laboratory). By mid-2020, CTR resistance started rising, reaching 69.1% in 2021 and 95.5% in 2022. Ciprofloxacin susceptibility persisted until 2022. The prevalence of the phenotype (AMP:   R, SXT:  R, CTR:  R, CIP:   S) surged from 8.5% in 2020 to 92.0% in 2022. 

The susceptibility of 283 *S. sonnei* isolates to AZ was tested by the disk diffusion method at one of the sentinel laboratories (HaEmek Medical Center) between 2017 and 2022. Only 5 (1.8%) of the 283 isolates were resistant to AZ. The proportion of resistance of these isolates for AMP, SXT, CTR and CIP were 44.9%, 66.6%, 39.2% and 1.7%, respectively. The other sentinel laboratories started routinely AST for AZ using the disk diffusion method only in 2023.

We examined potential host and environmental correlates such as sex, age, population group, and SES and the risk of acquired resistance by the *S. sonnei* isolates 2010–2022. None of the examined factors correlated with resistance to AMP, SXT or CIP. However, individuals with low SES had 4.9 times higher likelihood of CTR-resistant *S. sonnei* infection compared to those with high SES, as presented in Supplementary Table S5. Arabs had a lower risk of CTR-resistant isolates than Jews and others (OR: 0.7; 95% CI: 0.5–0.9; p = 0.011). Cases aged 15–24 and ≥25 years had an increased risk of CTR resistance.

We analysed the risk of acquired resistance by the *S. sonnei* isolates to the selected antimicrobial agents 2021–2022 when the sharp increase in AMR was observed. Arabs had a much lower risk of harbouring *S. sonnei* resistant to AMP (OR:  0.1; 95% CI: 0.03–0.1; p < 0.001), SXT (OR:  0.2; 95% CI: 0.1–0.3; p < 0.001) and CTR (OR: 0.1; 95%CI 0.03–0.2, p < 0.001) compared to Jews and others. None of the other host and environmental factors showed a similar association.

Another separate analysis on the cluster of *S. sonnei* isolates resistant to CTR in 2015 from the laboratory serving the population in the south of Israel revealed that the increased number and percentage of *S. sonnei* isolates resistant to CTR started by mid-2015 and lasted to the end of the year, with 76 of the 80 individuals with resistant isolates to CTR being younger than 15 years and with equal sex distribution. Arabs had a higher risk of harbouring *S. sonnei* resistant to CTR compared to Jews and others (OR = 9.4; 95% CI = 4.2–21.1; p < 0.001).

Between 2010 and 2022, 751, 297, 734 and 181 *S. flexneri* isolates were tested for resistance to AMP, SXT, CTR and CIP, respectively. The proportion of resistant isolates to AMP, SXT, CTR, and CIP was 60.2% (95% CI: 56.7–63.6), 50.8% (95% CI: 45.2–56.5%), 3.3% (95% CI: 2.2–4.7%) and 7.7% (95% CI: 4.5–12.3%), respectively. A total of 513, 136, 500 and 91 serotyped *S. flexneri* isolates were tested for resistance to AMP, SXT, CTR, and CIP, respectively. Of the *S. flexneri* 6 isolates tested, 47 (26.4%) of 178 were resistant to AMP, 47 of 50 to SXT, 2 (2.9%) of 69 to CTR and 1 of 18 to CIP. Of the *S. flexneri* 2a isolates tested, 108 (83.1%) of 130 were resistant to AMP, 10 of 27 to SXT, 2 (2.9%) of 69 to CTR and 5 of 26 to CIP.

## Discussion

We analysed trends in the incidence and characteristics of shigellosis in Israel in the past 25 years based on data collected through the SLBSN.

The mean incidence of shigellosis was ca 18 times higher than the overall 3.92 rate of culture-confirmed shigellosis per 100,000 population in 2016 in the US, reported by the active FoodNet population-based surveillance network for laboratory-diagnosed infections, including shigellosis [15] and ca 32 times higher than the 2.2 confirmed cases per 100,000 population reported by 30 European Union/European Economic Area (EU/EEA) countries in 2019 [16].

Since 2020, the introduction of PCR-based direct detection of *Shigella* in stool samples has approximately doubled the sensitivity of laboratory detection in Israel, consequently leading to a twofold or higher increase in notified disease incidence compared to culture-based methods. This enhanced sensitivity conferred by PCR-based detection is not unexpected for *Shigella*, given its fastidious nature and reduced growth yield in culture, especially when plating is delayed, allowing other bacteria to overgrow. Similar sensitivity increases have been reported in recent studies using the same commercial multiplex PCR systems [17] and are consistent also with values reported from case–control studies studying potential increases in the detection of carriage in non-diarrhoeal healthy individuals [18]. While PCR-based detection offers advantages, such as rapid detection and high sensitivity, it also has the potential drawback of false positives, as it cannot distinguish between viable pathogens, asymptomatic carriage or DNA remnants. However, in the context of the SLBSN, which deals with symptomatic individuals referred by physicians for differential laboratory diagnosis when *Shigella* is suspected, this drawback may not be critical [18].

We assume that the higher incidence of shigellosis in Jews and others than in Arabs in Israel, a setting of universal health insurance [19] likely reflects referral or healthcare utilisation patterns, health-seeking behaviour concerning diarrhoeal disease episodes in the community or a combination of these factors. The severity of the disease or onset earlier in infancy compared to the Jewish population as we show in the present paper might change this pattern, explaining the overrepresentation of the Arab population group in shigellosis associated with hospitalisations as previously reported [20].

The Arab population group displayed changing age-specific trends. This pattern may be attributed to differences in environmental and socioeconomic conditions, with Arab children living predominantly in rural areas, potentially enhancing exposure to more diverse *Shigella* transmission modes earlier in life, as also described for campylobacteriosis, another faecal-orally transmitted disease [21].

We observed an increase of *Shigella* infections in males aged 5 years in Jews and Arabs and a reverse excess of shigellosis in females in the older age groups, corroborating our previous observations [4]. The higher incidence of shigellosis in males at a very early age may suggest genetic and hormonal sex-associated factors playing a role [22], the higher risk ratio in adult females probably reflects the enhanced exposure of caregivers, mothers and grandmothers to *Shigella* when nursing young children suffering from shigellosis [4].

The secular trend of increase of *S. sonnei* relative weight in the aetiology of culture-confirmed shigellosis in Israel was driven by a decline in *S. flexneri* shigellosis incidence and is in line with a similar trend in countries undergoing economic development and improvements in sanitation and water quality [6,23]. It probably reflects the better adaptation of *S. sonnei* than *S. flexneri* to more restricted modes of transmission associated with specific *S. sonnei* determinants such as a unique O antigen, the presence of a capsule, a mechanism to displace other enteric bacteria, which all act in protection against environmental stresses [24].

Serotype 6 of *S. flexneri* was the most frequently isolated serotype during the 25-year surveillance, followed by *S. flexneri* 2a, 1b and 3a. These serotypes in the order 2a, 6, 3a and 1b were also the most common *S. flexneri* serotypes in children in LMIC [25], while 2a, 1b, 3a were most frequently isolated in the US and European high-income countries, mostly in MSM [15,16]. These serotypes are included in the configuration of *Shigella* multivalent vaccine candidates under clinical development [26].

*S. sonnei* drives cyclic shigellosis epidemics in Israel approximately every two years, particularly affecting those aged 1–4 years and areas served by M and D laboratories, including ultraorthodox Jewish communities in Jerusalem and Bnei Brak. Factors such as higher fertility rates, household crowding and poverty, along with lower median age and family income, contribute to these outbreaks [27]. Enhanced person-to-person transmission primarily occurs due to poor hand hygiene among toddlers, increased faecal contact during toilet training, and diapering practices in daycare or households [3,5].

The lower incidence in infants may result from reduced exposure in the first year and maternal antibody protection [28]. Neither the establishment nor the dynamics of persistent or dominant clones could explain the cyclic morbidity patterns as revealed by whole genome sequencing and phylogenetic analysis of 281 *S. sonnei* isolated in Israel between 2000 and 2012 [29]. Protection conferred by *S. sonnei* shigellosis against the same serotype within 2 years, likely mediated by specific IgG anti-*S. sonnei* lipopolysaccharide (LPS) antibodies, suggests that changes in population immunity drive cyclic peaks, rather than the epidemic agent itself [28].

There were two distinct seasonal decreases in the incidence rates of *S. sonnei*, one that coincided with the Passover vacation (during April) and the second, with the longer summer vacation between the end of July and September–October, when we assume that a significant decline in the person-to-person transmission of *S. sonnei* occurs with the closure of the daycare and preschool settings and reduction of mixing. The opposite trend of enhanced transmission and increase in incidence occurs during the full functioning of preschools between October and March and between April and July. *S. flexneri*, mostly occurring among Bedouins in the south of Israel, consistently increased in summer, suggesting the potential involvement of food-borne, waterborne, and fly-borne transmissions heightened by higher summer temperatures and the rural environment. These findings are important in guiding the design of future potential interventions to reduce the overall burden of shigellosis in Israel.

We detected a remarkable increase from 8.5% in 2020 to 52.0% in 2021 and to 92.0% in 2022 in the proportion of *S. sonnei* isolates simultaneously resistant to CTR, AMP and SXT. The isolates were part of the epidemic surge of *S. sonnei* shigellosis in 2022 after the lowest incidence of the disease during the peak of the COVID-19 pandemic in Israel 2020–2021 [30]. Resistance to CTR in *S. sonnei* isolates between 2021 and 2022 was mostly associated with the carriage of CTX-M-15 β-lactamase as determined at the Shigella National Reference Laboratory (personal communication, Analía V. Ezernitchi, Assaf Rokney, 5 May 2024). Most of the CTR-resistant isolates from 2021 and 2022 were susceptible to CIP. They were also susceptible to AZ, albeit the limited number of isolates tested. Interestingly, for the entire 2010–2022 period and the recent *S. sonnei* epidemic surge in 2022, the likelihood of being infected with an *S. sonnei* strain resistant to CTR was significantly higher in Jews and others than in Arabs while Arabs were more often infected with *S. sonnei* resistant to CTR in 2015 in the south of Israel without a further spread of the CTR-resistant *S. sonnei* strains.

The large increase in Israel in the proportion of *S. sonnei* isolates resistant simultaneously to CTR, AMP and SXT is alarming. This trend aligns with recent reports on *S. sonnei* MDR strains emerging in Asian countries, linked to travel to Asia, or involved in outbreaks occurring in communities or networks, such as, bisexual and MSM [7,8]. The situation in Israel is particularly of concern because *S. sonnei* with this AMR phenotype is currently intensively transmitted in the population and not restricted to a specific population group. Enhanced person-to-person transmission during the cyclic epidemics and more antimicrobials prescribed, increase the risk that *S. sonnei* strains could also acquire resistance to CIP through fixed chromosomal mutations and to AZ through horizontal transfer of various plasmids and AMR genes [10] and become resistant to at least one agent in all but one or two antimicrobial classes, defined as extensively drug-resistant (XDR). The recent increase in the proportion of *S. sonnei* isolates resistant to CIP, CTR and AZ identified in France between 2015 and 2021 [10] makes this scenario possible in Israel because of the enhanced transmission of shigellae [4,5].

The significant decrease in the number of *S. flexneri* isolates limits the power of following accurate annual changes in the AMR. Nevertheless, we could observe resistance rates to AMP and SXT like those previously reported in the same communities till 2008 [4] and consistently low resistance rates to ciprofloxacin and ceftriaxone. 

The present study has limitations. We are probably underestimating the actual burden of shigellosis in the community. An estimated multiplier of 25 was proposed to close the gaps in the surveillance steps in Israel [4]. This gap could be smaller today with the introduction of PCR-based detection of *Shigella* despite its drawbacks. Additionally, data on laboratory-confirmed cases that imply visits to a community clinic to be referred for laboratory testing could select for moderate to severe shigellosis compared to those not visiting a community clinic because of a milder disease. Conversely, we did not include *Shigella* isolates from hospitalised patients missing perhaps more severe cases of shigellosis requiring direct hospitalisation. There might also be a differential utilisation of medical services and health-seeking behaviour concerning diarrheal diseases in the Jewish and Arab population groups, possibly leading to a differential underestimation of the burden of shigellosis in Israeli Arabs. The findings on AMR were dependent on the various sentinel laboratories policy in antimicrobial susceptibility testing which have changed along the years. Nonetheless we believe that this did not affect the accuracy of the main trends in AMR of *Shigella* that we report here.

## Conclusions

The persistent high incidence of shigellosis mostly caused by *S. sonnei* and escalating antimicrobial resistance observed in *S. sonnei* continue to pose a significant public health challenge in Israel, notwithstanding the substantial advancements in basic sanitary services and infrastructure over recent decades. Intervention programs are needed, focusing on promoting personal hygiene, with a particular emphasis on handwashing, to reduce the transmission of shigellae during initial disease clusters. Additionally, once an efficacious and safe *S. sonnei* vaccine will be available, immunising children aged 1–4 years could be a strategic measure to disrupt the cycles of morbidity and substantially alleviate the burden of shigellosis in Israel.

## Supplementary Data

898000 Supplement
