# Supplementary material for: Twenty-five years of sentinel laboratory-based surveillance of shigellosis in a high-income country endemic for the disease, Israel, 1998 to 2022
Source: Euro Surveill. 2024 Aug 1;29(31):2400022. doi: 10.2807/1560-7917.ES.2024.29.31.2400022 (PMC11295440; doi:10.2807/1560-7917.ES.2024.29.31.2400022)

This supplementary material is hosted by *Eurosurveillance* as supporting information alongside the article 'Twenty-five years of sentinel laboratory-based surveillance of shigellosis in a high-income country endemic for the disease, Israel, 1998 to 2022', on behalf of the authors, who remain responsible for the accuracy and appropriateness of the content. The same standards for ethics, copyright, attributions and permissions as for the article apply. Supplements are not edited by *Eurosurveillance* and the journal is not responsible for the maintenance of any links or email addresses provided therein.

Table S1: Characteristics of cases of shigellosis identified through the sentinel laboratory-based surveillance network (SLBSN) (1998–2022), SLBSN population and the entire Israeli population in 2013.

		**Cases of shigellosis (1998–2022)**		**SLBSN Population (year 2013)**		**Israel population (year 2013)**	
**Variable**	**Category**	**N**	**%**	**N**	**%**	**N**	**%**
**Sentinel laboratory**	Haemek Medical Center (A)	2,238	4.5	522,039	21.0	NA	NA
	Clalit Haifa HMO (H)	7,417	15.0	730,998	29.4	NA	NA
	Maccabi Dan District HMO (D)	9,948	20.2	291,150	11.7	NA	NA
	Soroka Medical Center (S)	13,977	28.4	541,017	21.8	NA	NA
	Meuhedet HMO (M)	15,712	31.9	401,522	16.1	NA	NA
**Age group (years)**	0-4	32,121	65.7	266,598	10.7	833,400	10.3
	5-14	9,790	20.0	477,450	19.2	1,439,900	17.9
	15-19	614	1.3	207,117	8.3	624,600	7.7
	20-24	893	1.8	194,000	7.8	596,800	7.4
	25-29	1,431	2.9	174,780	7.0	582,100	7.2
	30-34	1,290	2.6	159,069	6.4	563,000	7.0
	35-44	1,323	2.7	309,284	12.4	1,033,200	12.8
	45-54	532	1.1	243,904	9.8	807,500	10.0
	55-64	439	0.9	216,344	8.7	729,600	9.1
	65-74	288	0.6	131,714	5.3	458,500	5.7
	75+	166	0.3	106,594	4.3	390,800	4.8
**Gender**	Male	24,365	50.0	1,233,705	49.6	3,991,300	49.5
	Female	24,406	50.0	1,253,250	50.4	4,068,100	50.5
**Population group**	Jews and Others	41,745	88.5	1,822,757	73.8	6,265,400	80.1
	Arabs	5,431	11.5	648,573	26.2	1,553,700	19.9
**Socioeconomic rank**	1-2	5626	13.5	NA	NA	1,039,514	12,9
	3-10	36031	86.5	NA	NA	7,005,517	87,1

NA=Not available

Table S2: Average annual incidence of shigellosis in the general surveillance population in 4 consecutive time periods within the 25 years of surveillance (1998-2022)

Period	Denominator	Cases	Incidence per 100,000	95%CI
1998-2004	13,570,374	15,452	113.8	112.09-115.6
2005-2011	15,817,584	12,704	80.3	78.94-81.7
2012-2019	21,027,177	16,910	80.4	79.21-81.6
2020-2022 (PCR positive)	7,680,872	3,620	47.1	45.61-48.6
2020-2022 (culture confirmed)	7,680,872	1,629	21.2	20.20-22.2

Table S3: Age-related male/female incidence rate ratios (IRR) of shigellosis in Jews and others and Arabs during 1998-2022

	Jews & Others								Arabs							
	Males			Females					Males			Females				
age groups	%	n	average annual incidence per 100,000	%	n	average annual incidence per 100,000	IRR	95%CI for IRR	%	n	average annual incidence per 100,000	%	n	average annual incidence per 100,000	IRR	95%CI for IRR
0	51.3%	670	158	48.7%	637	150	0.95	0.85-1.06	55.5%	464	229	44.5%	372	184	0.80	0.70-0.92
1-4	53.0%	13790	811	47.0%	12251	720	0.89	0.87-0.91	54.4%	1555	192	45.6%	1304	161	0.84	0.78-0.90
5-14	50.5%	4181	110	49.5%	4094	108	0.98	0.94-1.02	52.9%	611	34	47.1%	543	30	0.89	0.79-1.00
15-24	35.8%	443	13	64.2%	796	24	1.80	1.60-2.02	38.0%	68	5	62.0%	111	8	1.63	1.21-2.21
25-34	30.8%	738	25	69.2%	1659	56	2.25	2.06-2.45	36.3%	61	6	63.7%	107	10	1.75	1.28-2.40
35-44	35.5%	421	16	64.5%	765	29	1.82	1.61-2.05	31.5%	28	3	68.5%	61	7	2.18	1.39-3.41
45-54	37.0%	170	7	63.0%	289	12	1.70	1.41-2.05	35.1%	20	3	64.9%	37	6	1.85	1.07-3.19
55-64	29.9%	114	6	70.1%	267	13	2.34	1.88-2.92	34.0%	17	5	66.0%	33	9	1.94	1.08-3.48
65+	38.8%	158	6	61.2%	249	10	1.58	1.29-1.92	41.7%	15	5	58.3%	21	7	1.40	0.72-2.72
Total	49.6%	20685	95	50.4%	21007	96	1.02	1.00-1.04	52.3%	2839	38	47.7%	2589	35	0.91	0.86-0.96

Table S4: Resistance pattern of S. sonnei isolates (2010-2022) (corresponding to Figure 6)

	AM_Ampicillin			CIP_Ciprofloxacin			SXT_Sulfamethoxa_Trimeth			CTR_Ceftriaxone		
	N	% (R)	95% CI	N	% (R)	95% CI	N	% (R)	95% CI	N	% (R)	95% CI
2010	975	84.6%	82.3%- 86.8%	450	0.44%	0.1%- 1.4%	450	80.9%	77.1%- 84.3%	594	0.3%	0.1%- 1.1%
2011	339	74.9%	70.1%- 79.3%	141	0.71%	0.1%- 3.3%	141	97.2%	93.4%- 99.0%	219	2.3%	0.9%- 4.9%
2012	746	70.8%	67.4%- 74.0%	337	0.30%	0.0%- 1.4%	337	96.1%	93.7%- 97.8%	463	2.2%	1.1%- 3.8%
2013	696	88.6%	86.1%- 90.9%	268	0.37%	0.0%- 1.7%	270	97.0%	94.5%- 98.6%	467	2.8%	1.6%- 4.6%
2014	844	90.4%	88.3%- 92.3%	417	0.72%	0.2%- 1.9%	417	88.5%	85.2%- 91.3%	547	0.5%	0.2%- 1.5%
2015	357	67.2%	62.2%- 72.0%	131	2.29%	0.6%- 6.0%	131	98.5%	95.2%- 99.7%	267	30.3%	25.1%- 36.0%
2016	1602	28.3%	26.2%- 30.6%	979	0.41%	0.1%- 1.0%	979	87.0%	84.8%- 89.0%	1592	2.0%	1.4%- 2.8%
2017	670	15.5%	12.9%- 18.4%	317	0.95%	0.3%- 2.5%	479	93.7%	91.3%- 95.6%	670	1.0%	0.5%- 2.0%
2018	1028	13.0%	11.1%- 15.2%	854	1.05%	0.5%- 1.9%	1028	62.8%	59.9%- 65.8%	1028	1.2%	0.6%- 2.0%
2019	1221	10.2%	8.6%- 12.0%	1210	0.91%	0.5%- 1.6%	1220	53.2%	50.4%- 56.0%	1221	3.6%	2.7%- 4.8%
2020	403	14.4%	11.2%- 18.1%	403	0.25%	0.0%- 1.2%	403	67.5%	62.8%- 71.9%	402	8.5%	6.0%- 11.5%
2021	123	71.5%	63.1%- 79.0%	123	0.0%		123	74.0%	65.7%- 81.1%	123	69.1%	60.6%- 76.8%
2022	878	96.8%	95.5%- 97.8%	878	0.57%	0.2%- 1.2%	877	93.5%	91.7%- 95.0%	876	95.5%	94.0%- 96.8%

Table S5: Correlates of *S. sonnei* antimicrobial resistance (multivariate analysis)

CTR_Ceftriaxone						
		R	Total	OR	95%CI for OR	p-value
Age						
	0	36 (13.7%)	262	ref.	-	-
	1-4	672 (13.1%)	5129	1.257	0.821-1.925	0.293
	5-14	169 (10.2%)	1651	1.028	0.655-1.614	0.905
	15-24	50 (26.0%)	192	2.606	1.464-4.638	**0.001**
	25+	140 (16.4%)	855	1.686	1.057-2.689	**0.028**
Pop. group						
	Jews & others	872 (12.1%)	7215	ref.	-	-
	Arabs	166 (27.8%)	597	0.651	0.467-0.908	**0.011**
SES						
	1-2	206 (34.3%)	600	4.872	3.666-6.457	**<0.001**
	3-10	729 (12.7%)	5724	ref.	-	-

Supplementary Figure S1

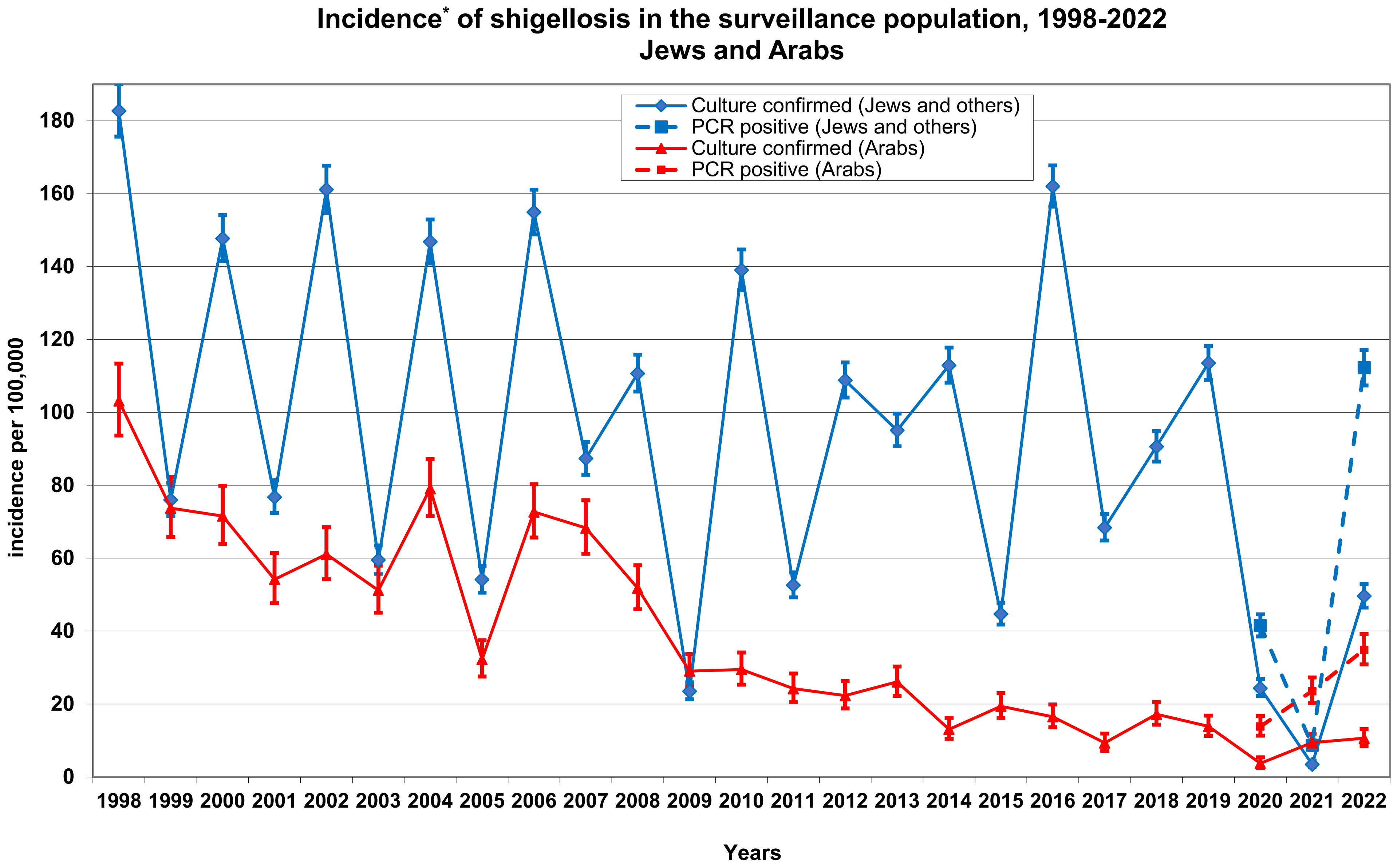

*Rates per 100,000 and 95% confidence intervals

Supplementary Figure S2

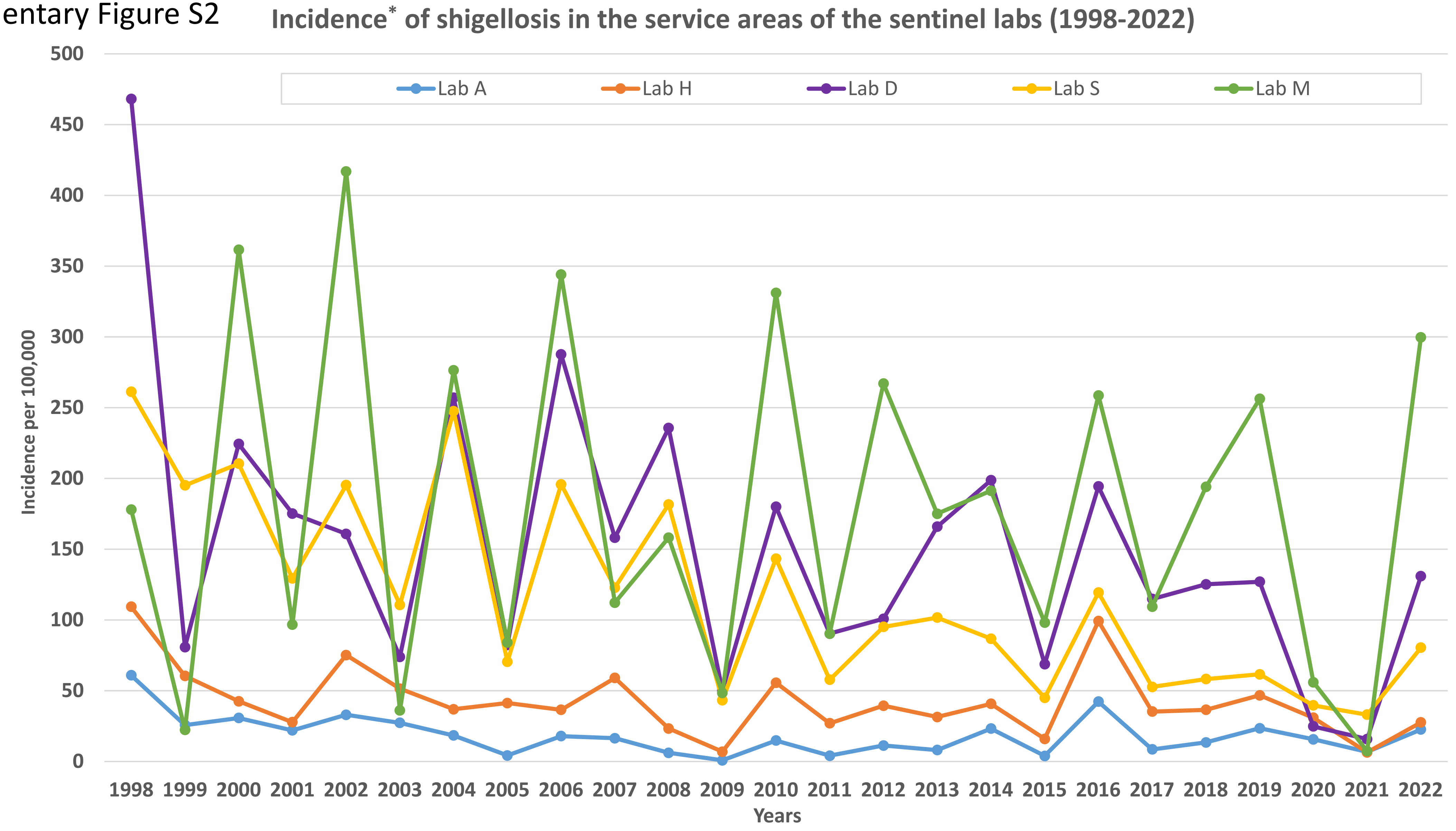

*Culture-proven between 1998-2019 and PCR-positivity-based between 2020 and 2022

Supplementary Figure S3

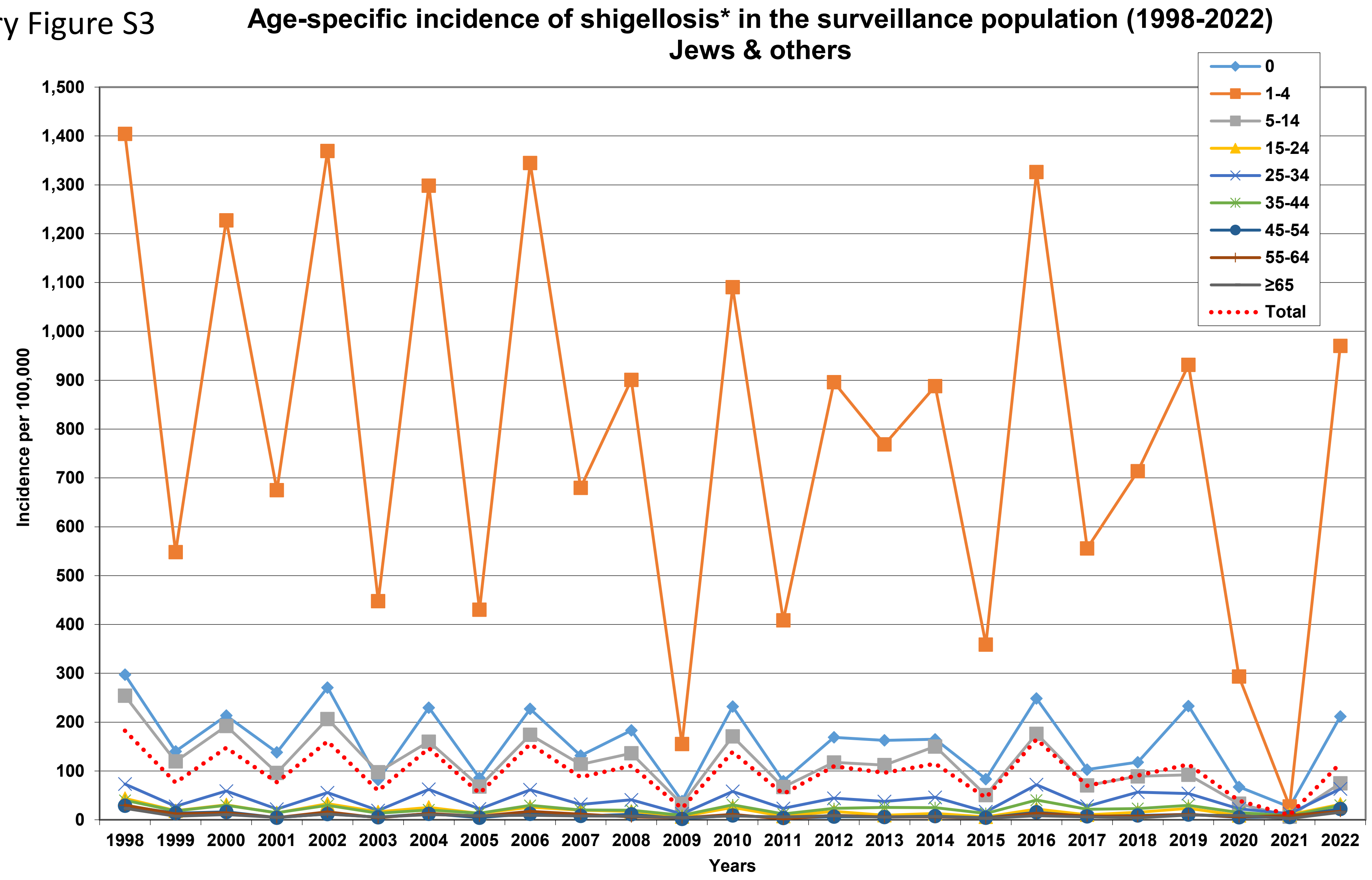

*Culture-proven between 1998-2019 and PCR-positivity-based between 2020 and 2022

Supplementary Figure S4

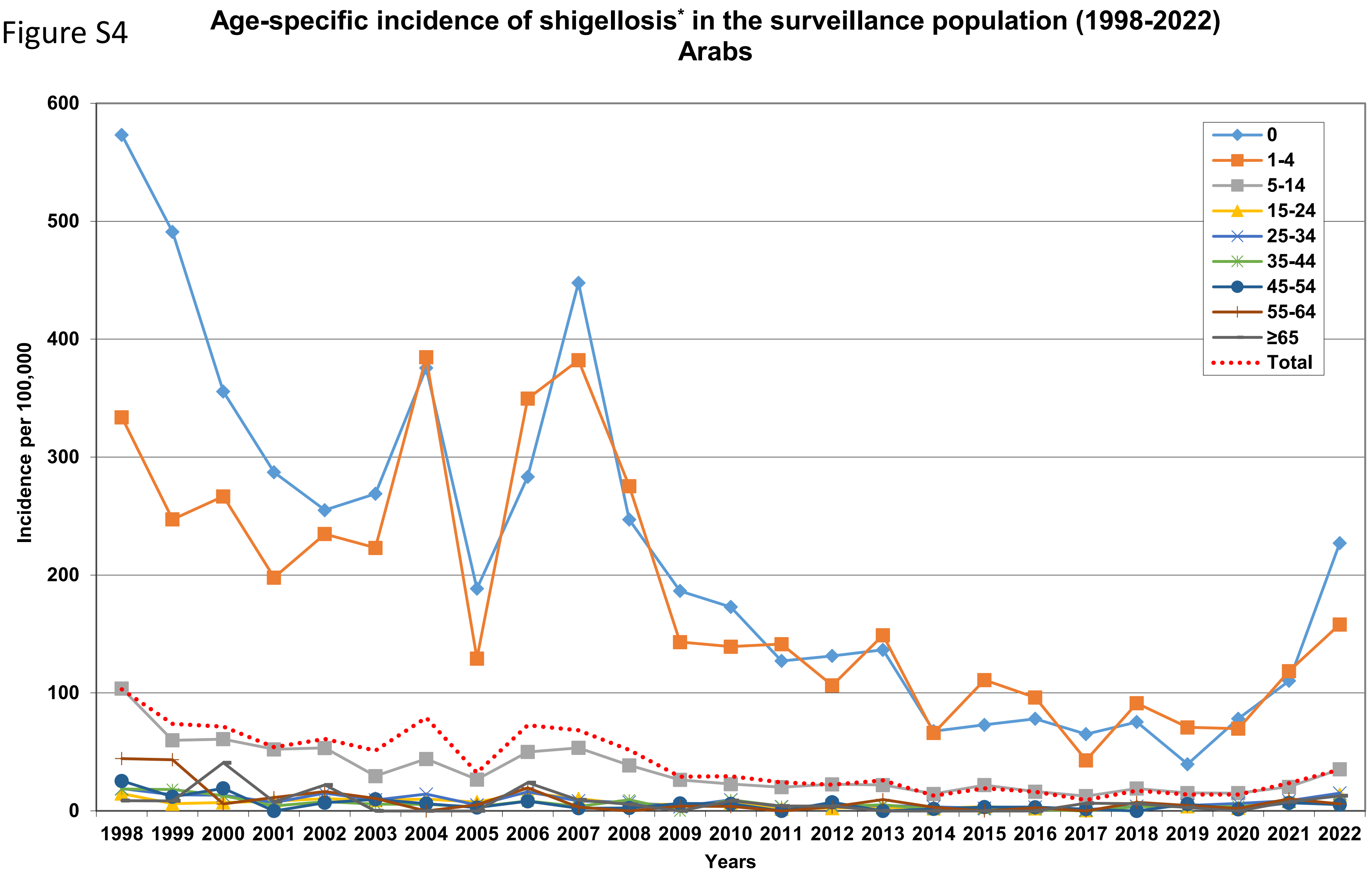

*Culture-proven between 1998-2019 and PCR-positivity-based between 2020 and 2022

Supplementary Figure S5: Secular trend in the annual incidence of shigellosis

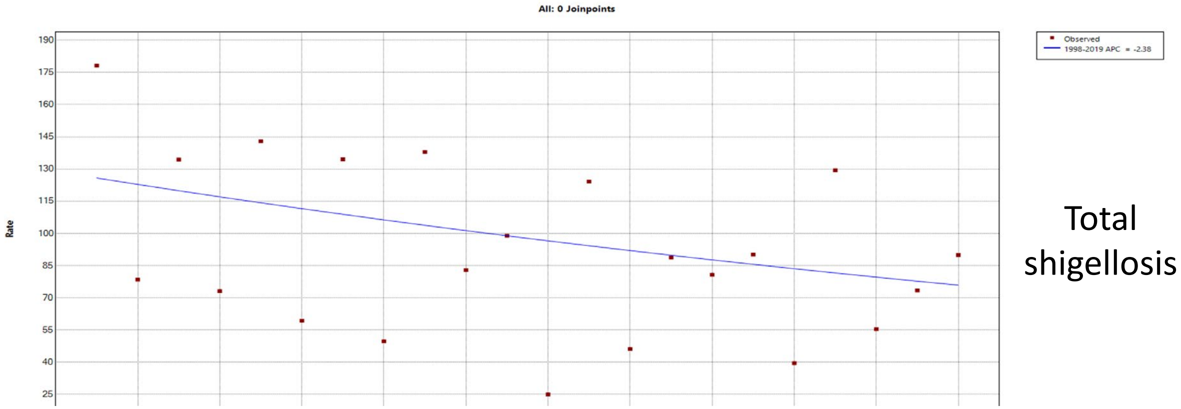

Total shigellosis

Graph | Data | Model Estimates | Trends | Model Selection | Comparison

Annual Percent Change (APC)

Segment	Lower Endpoint	Upper Endpoint	APC	Lower CI	Upper CI	Test Statistic (t)	Prob > |t|
1	1998	2019	-2.3779	-5.7909	1.1740	--	--

* Indicates that the Annual Percent Change (APC) is significantly different from zero at the alpha = 0.05 level.
-- Test Statistic and P-Value not available for the Empirical Quantile method. See Help to Learn More

Average Annual Percent Change (AAPC)

Range	Lower Endpoint	Upper Endpoint	AAPC	Lower CI	Upper CI	Test Statistic	P-Value
Full Range	1998	2019	-2.3779	-5.7909	1.1740	--	--

* Indicates that the AAPC is significantly different from zero at the alpha = 0.05 level.
-- Test Statistic and P-Value not available for the Empirical Quantile method. See Help to Learn More

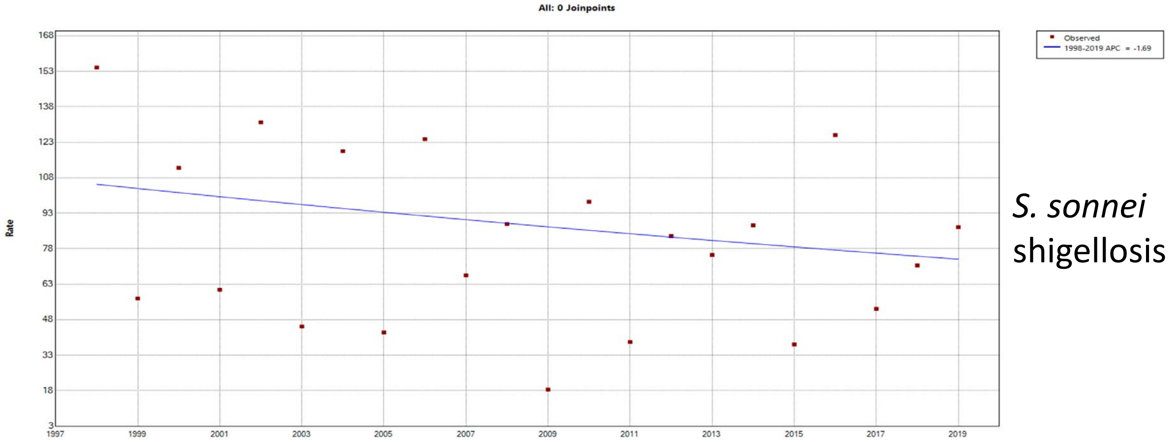

S. sonnei shigellosis

Graph | Data | Model Estimates | Trends | Model Selection | Comparison

Annual Percent Change (APC)

Segment	Lower Endpoint	Upper Endpoint	APC	Lower CI	Upper CI	Test Statistic (t)	Prob > |t|
1	1998	2019	-1.6897	-5.1613	2.1421	--	--

* Indicates that the Annual Percent Change (APC) is significantly different from zero at the alpha = 0.05 level.
-- Test Statistic and P-Value not available for the Empirical Quantile method. See Help to Learn More

Average Annual Percent Change (AAPC)

Range	Lower Endpoint	Upper Endpoint	AAPC	Lower CI	Upper CI	Test Statistic	P-Value
Full Range	1998	2019	-1.6897	-5.1613	2.1421	--	--

* Indicates that the AAPC is significantly different from zero at the alpha = 0.05 level.
-- Test Statistic and P-Value not available for the Empirical Quantile method. See Help to Learn More

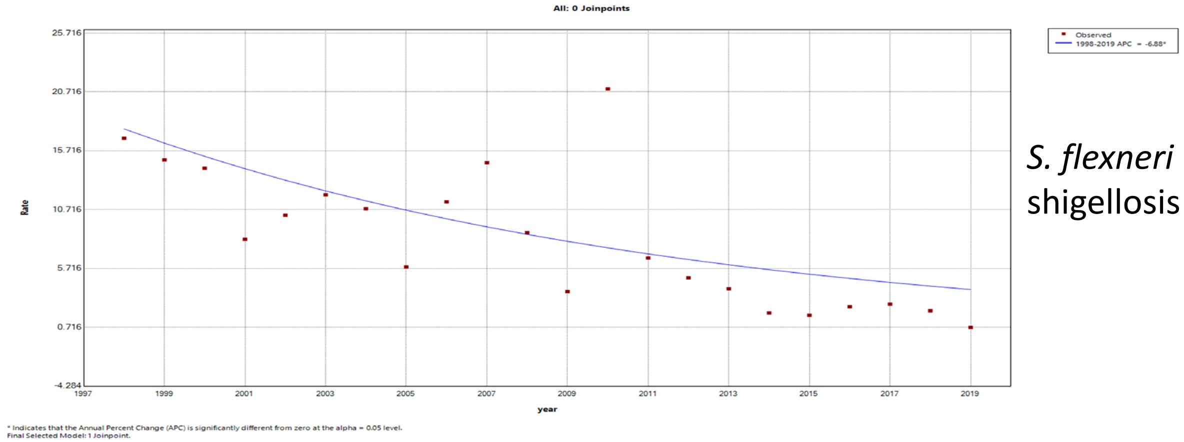

S. flexneri shigellosis

Graph | Data | Model Estimates | Trends | Model Selection | Comparison

Annual Percent Change (APC)

Segment	Lower Endpoint	Upper Endpoint	APC	Lower CI	Upper CI	Test Statistic (t)	Prob > |t|
1	1998	2019	-6.8815*	-11.0072	-2.5646	-3.2819	0.003728

* Indicates that the Annual Percent Change (APC) is significantly different from zero at the alpha = 0.05 level.

Average Annual Percent Change (AAPC)

Range	Lower Endpoint	Upper Endpoint	AAPC	Lower CI	Upper CI	Test Statistic~	P-Value~
Full Range	1998	2019	-6.8815*	-11.0072	-2.5646	-3.2819	0.003728

* Indicates that the AAPC is significantly different from zero at the alpha = 0.05 level.
~ If the AAPC is within one segment, the t-distribution is used. Otherwise, the normal (z) distribution is used. See Help to Learn More

Supplementary Figure S6

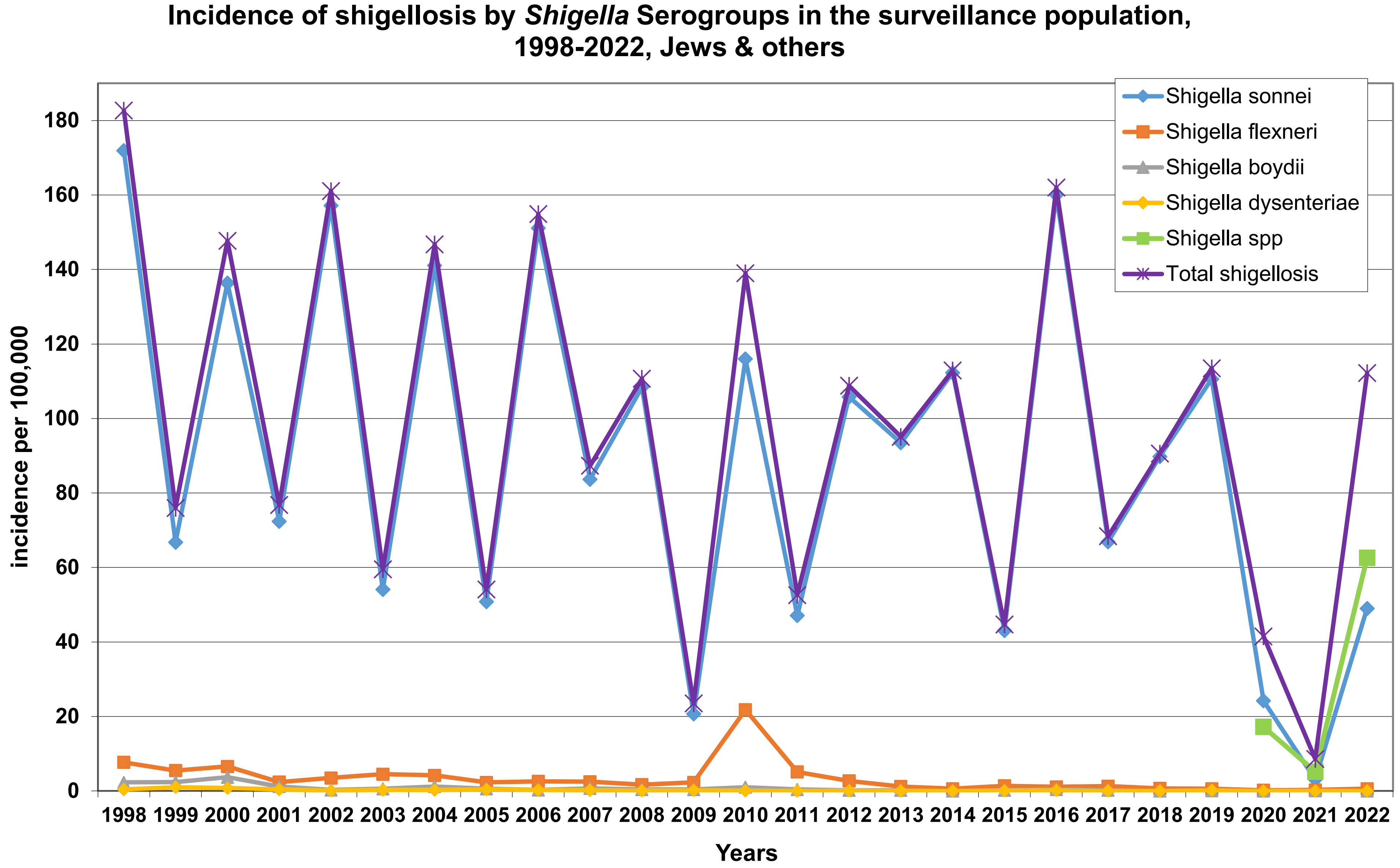

Total shigellosis = Before 2020, only culture-confirmed; from 2020 to 2022, based on ipaH positivity by PCR; Shigella spp. = positive by PCR but culture-negative

Supplementary Figure S7 **Incidence of shigellosis by *Shigella* Serogroups in the surveillance population, 1998-2022, Arabs**

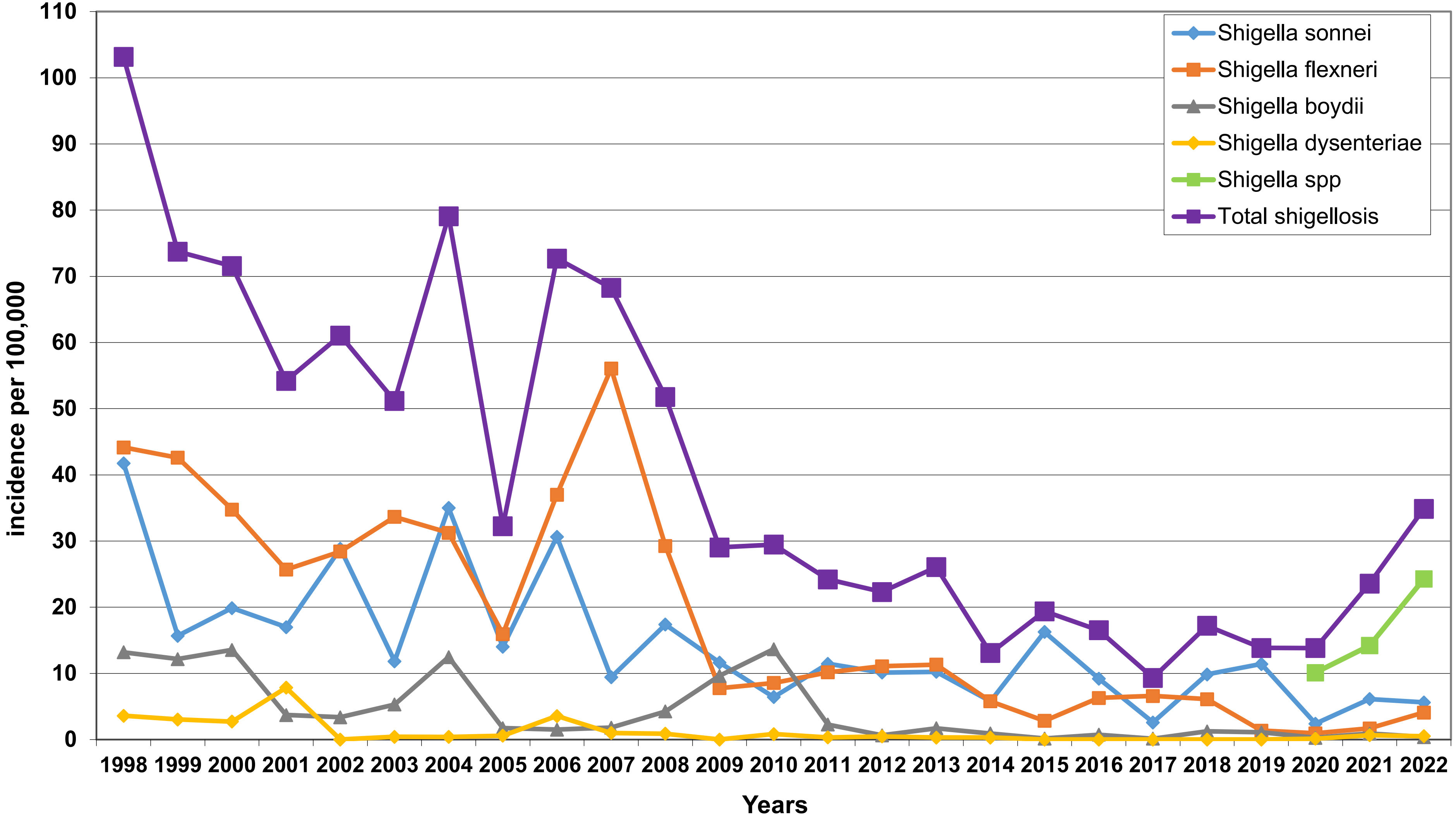

Total shigellosis = Before 2020, only culture-confirmed; from 2020 to 2022, based on ipaH positivity by PCR; Shigella spp. = positive by PCR but culture-negative

Supplementary Figure S8

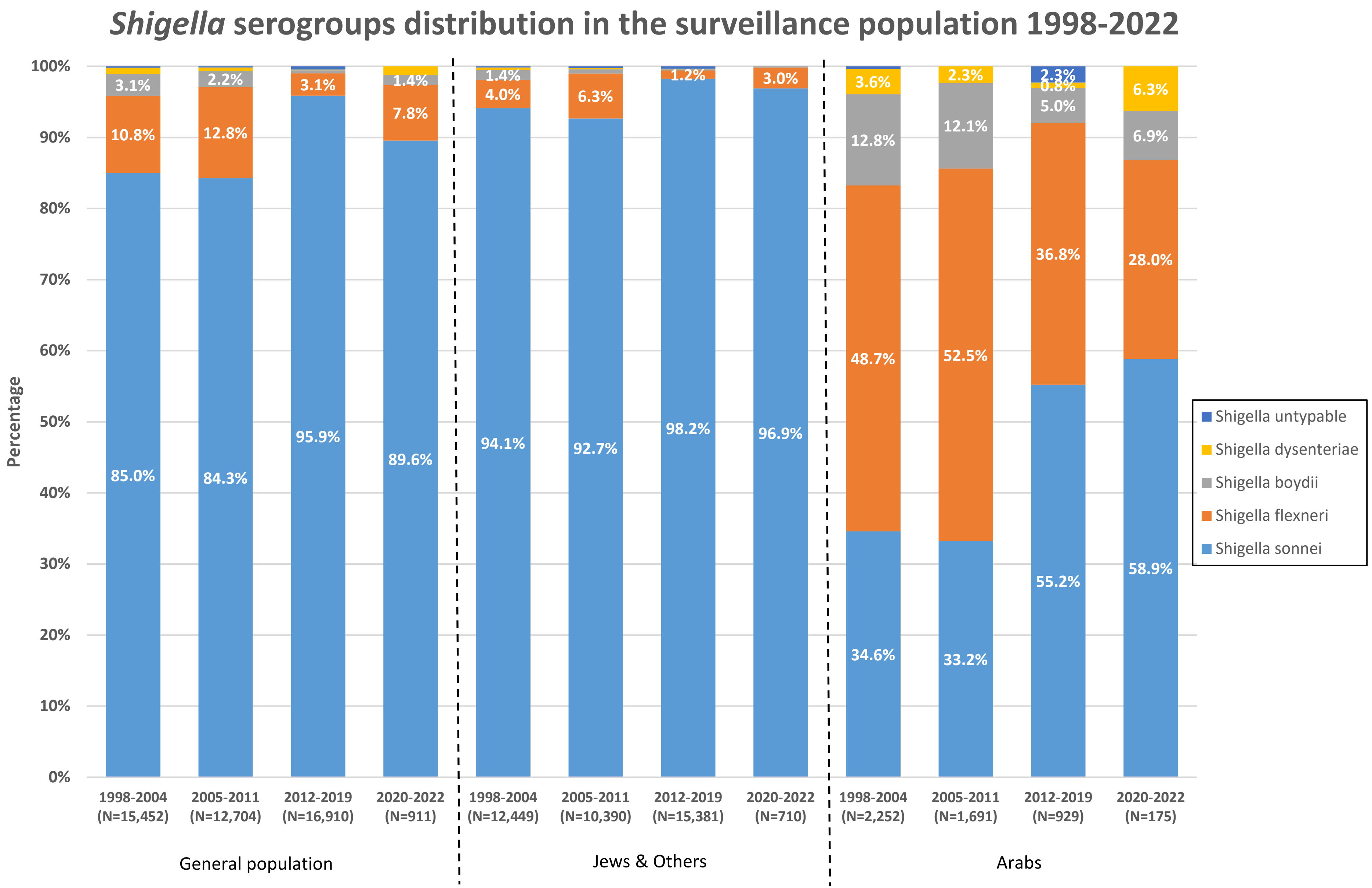

Supplementary Figure S9

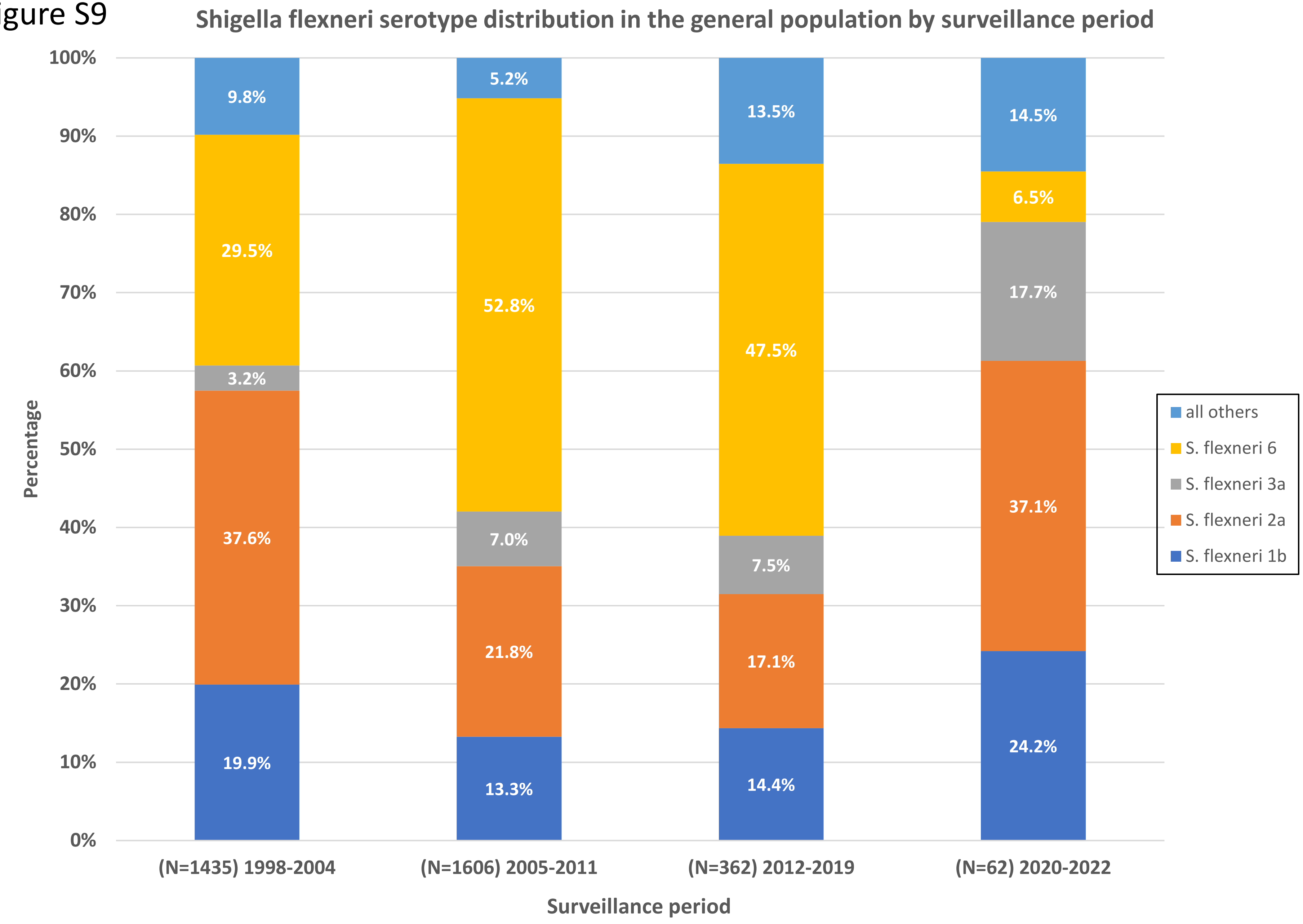

Supplement: Supplement [file 24-00022_COHEN_Supplement.pdf]
